# Health extension service utilization and associated factors in East Gojjam zone, Northwest Ethiopia: A community-based cross-sectional study

**DOI:** 10.1371/journal.pone.0256418

**Published:** 2021-08-19

**Authors:** Bewket Yeserah Aynalem, Misganaw Fikrie Melesse

**Affiliations:** Department of Midwifery, Debre Markos University, Debre Markos, Ethiopia; National Research Centre of Egypt, EGYPT

## Abstract

**Introduction:**

Health Extension Program is a preventive, promotive, and basic curative service targeting households to improve the health status of families with the effective implementation of 16 health extension packages. We, therefore, did this study to assess health extension package utilization and associated factors in the East Gojjam zone, Northwest Ethiopia.

**Methods:**

A community-based mixed cross-sectional study was conducted on households of East Gojjam Zone, from January 1 to April 30, 2020. A multistage sampling procedure was used to select 806 study participants in this study. We used EPI info version 7 for data entry and SPSS version 24 software for cleaning and analysis. Variables having a P-value of less than 0.25 in the bivariate logistic regression analysis were fitted into the multivariable logistic regression model. The 95% confidence interval of odds ratio was computed and a variable having P-value less than 0.05 in the multivariable logistic regression analysis was considered as statistically significant.

**Results:**

The study showed that 119 (14.8%) respondents have utilized health extension packages. Knowledge health extension package (AOR = 1.84, 95% CI: 1.22, 2.79), residence (AOR = 3.55, 95% CI: 1.99,6.33),visited health post(AOR = 1.63, 95% CI: 1.054,2.50), home visited by health extension worker (AOR = 1,68, 95% CI: 1.025,2.74) and involving in model family training(AOR = 2.10, 95% CI: 1.38,3.215) were significant factors for health extension service utilization.

**Conclusion:**

The magnitude of health extension service utilization was low since the Ethiopian government recommends 100% health extension service utilization coverage. Knowledge of health extension package, residence, health post-visit, home visit, and model family training were significant factors for health extension service utilization. So expanding the model family training and strict home-to-home visit especially in rural areas may increase the health extension package utilization.

## Introduction

Establishing an effective and responsive health service delivery system is an integral part of the overall development that aims to reduce poverty and achieve economic growth and development [1, 2]. The Health Extension Program (HEP) is a package of preventive, promotive, and basic curative services targeting households to improve the health status of families with their full participation [3]. HEPs include disease prevention and control, family health service, hygiene, and environmental sanitation, health education, and communication [1]. The former Frontline Health workers or traditional birth attendants (TBAs) are incorporated into the system by serving as volunteers that work under the supervision of the health extension worker (HEW) [4]. Thus, HEP will serve as a primary vehicle for prevention, health promotion, behavioral change communication, and basic curative care [5]. Globally many countries, especially developing countries are striving to achieve universal health coverage starting from the “Health for All” movement of 1978 by the World Health Organization (WHO) or the Almata declaration [6]. The Government of Ethiopia has implemented the Health Extension package (HEP) since 2003 to improve primary health coverage at the grassroots level [7] and also agreed to this movement and tried to address universal health coverage mainly primary health care (PHC) throughout the country [8].

The center for the HEP activity is a health post (HP) located in each smallest administrative unit of the country and is staffed by two female Health Extension Workers (HEWs) who receive one year’s training and a regular salary from the government [9, 10]. HEP was published to have public sector facilities provide a minimum standard of care that fosters an integrated service delivery approach [11]. And it delivers cost-effective basic services to all Ethiopians, mainly women, and children [12]. More than 75% of the health problem in Ethiopia is largely attributed to preventable communicable diseases and under-nutrition. The prevalence of these diseases is mainly due to poor socio-economic conditions, a low level of awareness about health, and inadequate health service delivery across the country [13]. Concerning this, the Ethiopian government designed a sound able plan to achieve universal access to primary health care by preparing a health sector development program (HSDP). This plan was aimed to address the service coverage problem of the health system through an accelerated expansion and strengthening of primary health care services [1, 12].

There are some factors associated with health extension service utilization in the previous study: these are knowledge of community on health extension service, age, occupation, community participation in the planning of health extension activities, and graduation of model family [18].

According to the Ethiopian Demographic and Health Survey of 2016, 38% of children under the age of five years suffered from stunting due to malnutrition; the Amhara region accounts for the highest proportion (46%) and the prevalence of water-borne and water-washed diseases were high. The Mini EDHS 2019 report also indicates under 5 mortality 30%, maternal mortality 412 per100,000 live births, and unmet need for family planning is 22% [14]. Therefore, this study aims to assess HEP utilization and its factors in East Gojjam Zone.

## Methods

### Study areas and period

East Gojjam zone is located in the Amhara region 300 km from Addis Ababa, a capital city of Ethiopia, and 265 km from Bihar Dar, the capital city of Amhara. It is bordered on the south by the Oromia Region, on the west by West Gojjam, on the north by South Gondar, and on the east by South Wollo. As the zonal health office report showed East Gojjam zone has a total population of 2,719,118 and 632,353 households. East Gojjam zone has also 21 Woreda, 480 Kebeles, 423 health posts, 102 health centers, 9 primary hospitals, and one referral hospital. The study was conducted from January 1, 2020, to April 30, 2020.

### Study design

A community-based cross-sectional study design was conducted.

### Study participants

The source population was all households who live in the East Gojjam zone. The study population was households in the east Gojjam zone during the study period in the selected Kebeles. Study participants age 18 and above who reside above six months in the study area were included and participants who were mentally ill during the data collection period were excluded.

### Sample size determination

The sample size was determined based on a single population proportion formula assumption. The expected proportion of HEP utilization (39%) from the previous study in Ethiopia Abuna Gindeberet, West Shoa Zone [15], and a 5% confidence limit (margin of error) was used.


$$\text{initialsamplesize}={\left(\text{Z}\frac{\text{a}}{2}\right)}^{2}*\frac{\text{p}(1-\text{p})}{\text{w}2}=1.96^{2}*\frac{0..39(1-0.39)}{({0.05)}^{2}}=366$$

Considering design effect 2 since it had two stages and the sample size was calculated as 366*2 = 732. Then the non-response rate was also considered to be 10% and 732*0.10 = 74. Then the final sample size was 732+74 = 806.

### Sampling techniques

A multistage sampling technique was used and firstly all the woreda found in the east Gojjam zone were listed in a frame. Then five out of the 21 woredas were selected by the lottery method. Again 4 kebeles, from Bibugn woreda, 5 kebeles from Debre Elias woreda, 5 kebeles from Dejen woreda, and 5 kebeles from Andede woreda were selected with the lottery method. The size of households consisting of the eligible population to be selected from each kebele was determined proportionally based on the size of the study units and the k^th^ value was computed for each selected kebele. Any single individual age 18 and above of the selected household was interviewed. In the case of absenteeism, after three repeated visits the next eligible household was included in the study.

### Study variables

#### Dependent variable

Health extension service utilization.

#### Independent variables

Age, marital status, religion, educational status, occupation, religion sex, residence, family size, knowledge, HP distance from home, HP visit, home visited by HEWs, model family training, model family graduation.

### Operational definitions

#### Health extension service utilization

Anyone who implements at least 75% of the national health extension program packages [16].

#### Satisfactory knowledge

Respondents who scored 75% and above of the HEPs knowledge questions [15].

#### Model households

Households that attended at least 75% of the training and implemented at least 75% of the HEP and given certificates of completion [10].

#### Health extension package

Is a package of preventive, promotive, and basic curative services targeting households to improve the health status of families with their full participation [3].

### Data collection and data quality control

To assure the data quality, data were collected with face-to-face interviews by three trained diploma nurses after two-day data collection training was given to them together with three BSc holder supervisors. The questionnaire was structured and pre-tested which was first prepared in English and translated to local (Amharic) language and then again translated back to English. A pretest was conducted on 40 households of the sample size other than the study area and the necessary correction on the tool was employed accordingly.

### Data processing and analysis

Epi Info version 7 software was used for data entry and SPSS version 24 for used for analysis. Bivariate logistic regression was employed to identify an association between independent and dependent variables. Variables having a P-value of less than 0.25 in the bivariate logistic regression analysis were fitted into the multivariable logistic regression model. The 95% confidence interval of odds ratio was computed and a variable having P-value less than 0.05 in the multivariable logistic regression analysis was considered as statistically significant.

### Ethical clearance

Ethical clearance was obtained from the research committee (Institutional Research Ethics Review Committee) of Debre Markos University with ethical approval number HSC/R/C/Ser/Co/341/06/12 and was submitted to the East Gojjam zone health bureau. Ethical clearance and formal letters were also obtained from the Debre Markos University and were submitted to the East Gojjam zone health bureau and permission was obtained. Finally, written informed consent was also obtained from each study participant in the sampled households.

## Results

### Socio-demographic characteristics

All 806 study participants responded to the questionnaire, giving a response rate of 100%. All of the participants were Amhara in ethnicity and 758 (94.1%) of the participants are orthodox Christian. Five hundred thirteen (63.6%) were living in a rural area with agriculture as a source of income (Table 1).

**Table 1:** Sociodemographic characteristics of respondents (n = 806) in East Gojjam Zone, Northwest Ethiopia, 2019.

Variable	Frequency	Percent
**Age(**in years**)**		
18–24	30	3.7
25–39	357	44.3
≥40	419	52
**Marital status**		
Single	48	5.9
Married	696	86.4
Widowed	20	2.5
Divorced	42	5.2
**Religion**		
Orthodox	758	94.1
Muslim	38	4.7
Protestant	10	1.2
**Educational status**		
No formal education	485	60.2
Primary education	126	15.6
Secondary education	90	11.2
College and above	105	13
**Occupation**		
Housewife	46	5.7
Self-employee (doing own small business)	138	17.6
Private employee(salaried in the nongovernmental sector)	44	5.5
Government employee	65	8.1
Farmer	513	63.6
**Residence**		
Rural	513	63.6
Urban	293	36.4
**Source of income**		
Agriculture	513	63.6
Other*	293	36.4
**Sex**		
Male	152	18.9
Female	654	81.1
**Family size**		
1–4	316	39.2
4^+^	490	60.8

### Service-related characteristics

Less than half (37%) of the study participants have transport access to the health post. Two hundred-one (90.5%) of the participants got a good approach from HEWs. Again around 208 (93.7%) participants got participants needed (Table 2).

**Table 2:** Service related issues among respondents (n = 806) in East Gojjam Zone, Northwest Ethiopia, 2019.

Variable	Frequency	Percent
Transport access to the health post		
Yes	298	37
No	508	63
Visited health post		
Yes	222	27.5
No	584	72.5
Reasons for visiting health post		
For antenatal care	116	48.7
For family planning	91	38.2
For other services	31	13.1
health extension worker approach		
Good	201	90.5
Bad	21	9.5
Returned without getting the service needed		
Yes	14	6.3
No	208	93.7
Reasons returned without getting the service		
Absence of health extension workers	8	57.1
Unavailability of the service	6	42.9
Home visited by a health extension worker		
Yes	544	67.5
No	262	32.5

### Knowledge and modeling related characters

Among the respondents, only 311(38.6%) had satisfactory knowledge of HEPs. About 259(32.1%) of respondents have participated in model family training of HEPs. Of these 249 (96.1%) had been graduated. One hundred ten (44.2%) of these had 1–2 years, 122(49%) had 3 years and 17(6.8%) had greater than 4 years after model family graduation.

### Utilization of the health extension package and its associated factors

One hundred nineteen (14.8%) of the study population have utilized health extension packages [95% CI: 12.4, 17.2] (Table 3). Bivariate logistic regression was used to identify an association between independent and outcome variables. Variables with P-values <0.25 in binary logistic regression (educational status, residence, knowledge, health post-visit, home visited by HEW, and model family training) continued to be fitted into the multivariable logistic regression.

**Table 3:** Utilization of health extension package (n = 806) in East Gojjam Zone, Northwest Ethiopia, 2019.

Package	Frequency	percent
A: **Hygiene and sanitation**		
Proper and safe excreta disposal		
Yes	366	45.4
No	440	54.6
Proper and safe solid and liquid management		
Yes	268	33.3
No	538	66.7
Personal hygiene		
Yes	310	38.5
No	496	61.5
Water supply and hygiene		
Yes	142	17.6
No	664	82.4
Proper and safe home environment		
Yes	469	58.2
No	337	41.8
Insects and rodents control		
Yes	592	73.4
No	214	26.6
Food hygiene		
Yes	471	58.4
No	335	41.6
B: **Communicable disease prevention and control**		
HIV/AIDS and TB prevention and control		
Yes	610	75.7
No	196	24.3
Malaria prevention and control		
Yes	461	57.2
No	345	42.8
First aid		
Yes	77	9.6
No	729	90.4
Youth reproductive health care		
Yes	179	22.2
No	627	77.8
Child and maternal health care		
Yes	481	59.7
No	325	40.3
Maternal and child nutrition		
Yes	387	48
No	419	52
Immunization		
Yes	583	72.3
No	223	27.7
Family planning		
Yes	518	64.3
No	288	35.7
C: **Communication**		
Communication and health education		
Yes	577	71.6
No	229	28.4
**Over all utilization**		
Yes	119	14.8
No	687	85.2

After controlling the effect of other variables with multivariable logistic regression analysis, residence [AOR: 3.549(95% CI: 1.99, 6.33)], knowledge [AOR: 1.84(95% CI: 1.216, 2.796)] health post-visit [AOR: 1.63(95% CI: 1.054, 2.50)], home visited by HEW [AOR: 1.68(95% CI: 1.03,2.740)] and Graduated from model family training [AOR: 2.10(95% CI: 1.38,3.22)] were significant factors for health extension package utilization (Table 4).

**Table 4:** Bivariable and multivariable analysis of factors associated with utilization of health extension package in East Gojjam Zone, Northwest Ethiopia, 2019.

Variable	Utilized		Crude OR[95%CI]	AOR[95%CI]
	No	Yes		
**Age**				
18–24	26	4	1	
25–39	307	50	0.94 (.35, 3.16)	
≥40	354	65	1.194(.403,3.53)	
**Marital status**				
Single	41	7	1	
Married	590	106	1.50 (.46,2.41)	
Widowed	18	2	.651(.12, 3.44)	
Divorced	38	4	.617(.17,2.27)	
**Religion**				
Orthodox	644	114	1	
Muslim	35	3	.48 (.15,1.60)	
Protestant	8	2	1.412(.29, 6.74)	
**Educational status**				
No formal education	434	51	**1**	1
Primary education	99	27	**3.32 (1.39,3.88)**	1.126(.60,2.09)
Secondary education	74	16	**1.84 (.99, 3.39)**	.64 (.302,1.39)
College and above	80	25	**2.66(1.56,45)**	.774(.370,1.615)
**Sex**				
Female	564	90	1	
Male	123	29	1.48(.93, 2.35)	
**Residence**				
Rural	466	47	**1**	**1**
Urban	221	72	**3.23 (2.16, 4.82)**	**3.55(1.99,6.33)** **
**Family size**				
1–4	269	47	1	
Above 4	418	72	.99(.66,1.47)	
**Knowledge**				
Unsatisfactory	437	58	**1**	**1**
Satisfactory	250	61	**1.84(1.24, 2.72)**	**1.84 (1.22, 2.79)** **
**Visited health post**				
No	511	73	**1**	**1**
Yes	176	46	**1.83 (1.22, 2.75)**	**1.63(1.05, 2.50)**
**Home visited by HEW**				
No	237	25	**1**	**1**
Yes	450	94	**1.98(1.24,316)**	**1.68(1.03, 2.74)**
**Participated in model family training**				
No	484	63	**1**	**1**
Yes	203	56	**2.12(1.43,3.15)**	**2.10 (1.38, 3.22)**
**Graduated from model family training**				
No	9	1	1	
Yes	194	55	2.55 (.32, 20.56)	

** P value< 0.001, 1reference,

*housewife self-employee, private employee, and government employee.

## Discussion

The study finding of health extension package utilization (14.8%) in the current study was higher than the study done in Hosanna, Hadya Zone, Southern Ethiopia (2.352%) [17]. The possible explanation for this difference might be the difference in socio-economic status and cultural aspects and differences in the level of awareness about health extension packages.

But the result of this study is lower than the study conducted in Abuna Gindeberet district, West Shoa Zone, Ethiopia (39%) [18]. The possible reasons for this difference could be the difference in study area coverage. This study was done at a zonal level that covers a wide area that might have great variation in health extension package utilization across the zone and leads to low coverage of HEP utilization. Again this finding is lower than the study done in Gulelle sub-city Administration, Addis Ababa, Ethiopia (86%) [15]. The possible explanation for this difference can be the study setting, socio-economic status of the community, and living standards. Our study was conducted in areas including the rural community which is unthinkable to be comparable with a community living in Addis Ababa. People in the Gulelle sub-city administration utilize the health extension packages on their own since they understand the importance of these packages whereas the rural community considers the importance of these packages is not for them rather either for the health extension workers or for the government. The other possible reason may be a limitation in basic infrastructures in rural communities relative to the Gulelle sub-city administration community.

As shown in this study, residence was one of the significant factors for the utilization of health extension package utilization. Urban dwellers were 3.55 times more likely to utilize the health extension packages as compared with rural households (AOR = 3.55, 95% CI: 1.99, 6.33). This finding was supported by the studies done in Kombolcha town district, East Hararghe Zone, Eastern Ethiopia, and Esera district of Southern Ethiopia [19, 20]. The possible explanation for this might be urban residents might have higher information access about health extension packages than the rural dwellers and there might have infrastructure assess to utilize these package.

Participants with a satisfactory knowledge of health extension packages were 1.84 times more likely to utilize these packages than respondents with their counterparts (AOR = 1.84, 95% CI: 1.22, 2.79). This result is supported by other studies conducted in Ethiopia as nationwide, Abuna Gindeberet District, West Shoa Zone, Ethiopia, and Hadya Zone, Southern Ethiopia [17, 18, 21, 22]. knowledge is the entry point for any activity, so respondents who know the health extension package could utilize those packages than their counterparts. Again participants who got the opportunity to know about health extension packages may have an effort to utilize it than those who do not have.

The other variable which was significantly associated with the health extension service utilization was health post-visit. Respondents who had visited the health post were 1.63 times more likely to utilize the health extension packages than those who did not visit the health post (AOR = 1.63, 95% CI: 1.054, 2.50). This finding was supported by a nationwide finding of Ethiopia [23]. The possible explanation for this difference might be as the respondents visit the HP they may have the opportunity to be familiar with the HEWs and get enough information about the HEP utilization than those who did not visit. When the respondents visit the HP, they can access the HEWs to talk frankly about HEP utilization with the aid of demonstration in addition to the home to home visit than those who do not visit. Visiting the HP by itself is using the HEP like ANC follow-up, HIV/AIDS counseling, family planning access, malaria prophylaxis, and prevention access, getting health education, and other packages. Therefore, respondents who visit HP may easily utilize HEP than those who did not.

Respondents from the household which is visited by the HEW were 1.676 times more likely to utilize the HEP than from not visited households (AOR = 1.68, 95% CI: 1.03, 2.74). This result was supported by a study done in Akaki district, Addis Ababa, and nationwide studies conducted in Ethiopia [23–25]. Visiting the households by the HEWs is the key factor for HEP utilization by the community. During the HEWs home to home visit, they perform lots of activities concerning HEP like registering pregnant women with their expected date of delivery, proper utilization of latrine, kitchen, insect acid-treated net, family planning, diagnose ill individuals in the household, and refer to health institution, provide health education and proper disposal of waste materials. So study participants whose houses are visited by the HEWs can utilize HEP than which is not visited.

Participants who had been involved in model family training were 2.10 times more likely to implement the health extension packages than those who had not participated (AOR = 2.10, 95% CI:1.377, 3.22). This result was supported by a study conducted in Nefas Silk Lafto Sub-city, Addis Ababa, Ethiopia [26]. The possible explanation might be involving the model family training regarding the health extension package increases awareness about these packages utilization than those who did not participate. The other reason could be when participants are involved in the model family training; they may have inspired to utilize the HEP than those who do not participate.

## Conclusion

The magnitude of health extension service utilization was low as compared to other studies. Knowledge, residence, health post-visit, home visit, and model family training were significant factors for health extension service utilization. So expanding the model family training and strict home-to-home visit especially in rural areas may increase the health extension package utilization.

## Limitation and strength

The strength of this study was the data source which was primary data collected directly from the community that makes it more accurate and representative for the study population. Difficulty of data collection from the community due to COVID-19 in terms of cost (availing sanitizer, face masks and transport) and the nature of study design (cross sectional study design) which cannot show the cause and effect relationship between the independent and the outcome variables were the limitation of this study.

## Supporting information

S1 Dataset (SAV)

S1 Questionnaire (DOCX)
