# Supplementary material for: Health extension service utilization and associated factors in East Gojjam zone, Northwest Ethiopia: A community-based cross-sectional study
Source: PLoS One. 2021 Aug 19;16(8):e0256418. doi: 10.1371/journal.pone.0256418 (PMC8376075; doi:10.1371/journal.pone.0256418)
Supplement: S1 Questionnaire — (DOCX) [file pone.0256418.s002.docx]

**English Questionnaire**

**I: Socio demographic characterstics**

| **S.No** | **Questions** | **Responses** | **Remark** |
| --- | --- | --- | --- |
| 101 | Age? | ___years |  |
| 102 | Sex | 1. female 2. male |  |
| 103 | Marital status | 1. single 2. married 3. widowed 4. divorced |  |
| 104 | Family size(in number) | __________ |  |
| 105 | Religion | 1. Orthodox 2. Muslim 3. Protestant 4. Catholic 5. Other______ |  |
| 106 | Ethnicity | 1. Amhara 2. Oromo 3. Gurage 4. Tigre 5. Others |  |
| 107 | Source of income | 1. Agriculture 2. Others source_____ |  |
| 108 | Educational status | 1. No formal education 2. Primary education 3. Secondary education 4. College level and above |  |
| 110 | Residence | 1. Rural  2. Urban |  |

**II:Service related questions**

| **S.No** | **Questions** | **Responses** | **Remark** | |
| --- | --- | --- | --- | --- |
| 201 | Is there transportation access to go to the health post? | 1. Yes  2. No |  |  |
| 202 | Have you visited the HP? | 1. Yes  2. No |  |  |
| 203 | If you yes for Q 203, for what purpose? | 1. ANC Follow up  2.Family planning  3.other_____ |  |  |
| 204 | How the HEWs approach with you? | 1. good 2. bad |  |  |
| 205 | Have you ever returned back to your home without getting the service you need? | 1. Yes  2. No |  |  |
| 206 | If yes for Q206, why? | 1. In availability of services  2. In availability of HEWs |  |  |
| 207 | Have HEWs visited your home | 1. Yes 2. No |  |  |

**III:Knowledge and modeling related questions**

| S.No | Questions | Responses | Remark |
| --- | --- | --- | --- |
| 301 | Have you heard about HEP? | 1. Yes 2. No |  |
| 302 | Proper and safe excreta disposal is a component of HEP | 1. Yes 2. No |  |
| 303 | Proper and safe solid and liquid management is a component of HEP | 1.Yes  2. No |  |
| 304 | Personal hygiene is a component of HEP | 1.Yes  2. No |  |
| 305 | Water supply and hygiene is a component of HEP | 1.Yes  2. No |  |
| 306 | Proper and safe home environment is a component of HEP | 1.Yes  2. No |  |
| 307 | Insects and rodents control is a component of HEP | 1.Yes  2. No |  |
| 308 | Food hygiene  is a component of HEP | 1.Yes  2. No |  |
| 309 | HIV/AIDS and TB prevention andcontrol is a component of HEP | 1.Yes  2. No |  |
| 310 | Malaria prevention and control is a component of HEP | 1.Yes  2. No |  |
| 311 | First aidskillis a component of HEP | 1.Yes  2. No |  |
| 312 | Youth reproductive health care is a component of HEP | 1.Yes  2. No |  |
| 313 | Child and maternal health careis a component of HEP | 1.Yes  2. No |  |
| 314 | Maternal and child nutrition is a component of HEP | 1.Yes  2. No |  |
| 315 | Immunization is a component of HEP | 1.Yes  2. No |  |
| 316 | Family planning is a component of HEP | 1.Yes  2. No |  |
| 317 | Communication and health education is a component of HEP | 1.Yes  2. No |  |
| 318 | Is there any family member ever participated in the model family training? | 1. Yes  2. No |  |
| 319 | Are you graduated as model family? | 1. Yes  2. No |  |
| 320 | If yes for Q 319, how long is it after graduation of HEP? | 1. 1–2 years  2. 3 years  3. ≥4 years |  |

**IV: Utilization of health extension service packages.**

| 1. **Hygiene and sanitation** | | | | | | | | |
| --- | --- | --- | --- | --- | --- | --- | --- | --- |
| **S.No** | | | **Questions** | | | **Responses** | | **Remark** |
| **501** | **Excreta disposal** | | 1.1 Is there any toilet facility available in your home? | | | 1. Yes  2. No | |  |
|  |  |  | 1.2 What kinds of to ilet facility do your members o f ho useho ld use? | | | 1. Private latrine 2. Public latrine | |  |
|  |  |  | 1.3 Do you always use your toilet? | | | 1. **Yes** 2. **No** | |  |
|  |  |  | 1.4Is there hand-washing facilities attached with toilet? | | | 1. Yes  2. No | |  |
| **502** | **Personal hygiene** | | - 1. Do you wash your hands before preparing food? | | | 1. Yes  2. No | |  |
|  |  |  | 2.2 Do you wash your hands before feeding? | | | 1. Yes  2. No | |  |
|  |  |  | 2.3 What do you usually use to wash your hands? | | | 1. Soap  2. Ash  3. Nothing is used | |  |
| **503** | **Proper and safe solid and liquid management** | | 3.1 Where do you dispose your wastes? | | | 1. Thrown into open pit    2. Thrown in to pit with cover  3. Thrown anywhere  4. Burning  5. composting | |  |
|  |  |  | 3.2 Do you have drainage for the liquid waste disposal | | | 1. Yes  2. No | |  |
| **504** | **Water supply measure** | | 4.1 from which water source always your family use | | | 1. Pipe  2. River  3. other___ | |  |
|  |  |  | 4.2 do you always use water cleaning method/s for unclean water before use? | | | 1. Yes 2. No | |  |
|  |  | | 4.3 What type of container do you usually use to store drinking water? | | | 1.Pot  2. Jerican  3.Bucket  4.Barrel | |  |
| **505** | **Healthy home environment** | | 5.1 do you have a separate kitchen? | | | 1. Yes  2. No | |  |
|  |  |  | 5.2 Haveyou constructed a kitchenware shelf? | | | 1. Yes  2. No | |  |
|  |  | | 5.3 Is your home separatedfromdomestic animals room? | | | 1. Yes  2. No | |  |
| **506** | **Insects and rodents control** | | 6.1 What measures do you take for prevention of rodents in your home? | | | 1. usebiological methods  2. use mousetrap  3.use chemical poisons | |  |
|  |  |  | 6.2 How can you prevent insects? | | | 1. using insecticides  2. Boiling the clothes  3.By using mechanical method | |  |
| **507** | **Food hygiene and safety** | | 7.1 Are foods kept covered? | | | 1. Yes  2. No | |  |
|  |  |  | 7.2 do you usually use food preservation method/s? | | | 1. Yes  2.No | |  |
| 1. **Communicable disease prevention and control** | | | | | | | |  |
| **508** | | **HIV/AIDS and TB** | | 8.1 Have been educated about HIV by HEWs | 1. Yes  2. No | | |  |
|  |  |  |  | 8.2 Have you been counseled for HIV test? | 1. Yes  2. No | | |  |
|  |  |  |  | 8.3 Have you been tested for HIV? | 1. Yes  2. No | | |  |
|  |  |  |  | 8.4 Have you ever been educated about Tuberculosis by HEWs? | 1. Yes  2. No | | |  |
|  |  |  |  | 8.5 What do you do when ther e is cough for more than two weeks? | 1. Visit Health HP  2. Take traditional medicine  3. dong no thing | | |  |
| **509** | | **Malaria** | | 9.1 Is there Malaria in your area? | 1. Yes  2. No | | |  |
|  |  |  |  | - 1. If yes, do have used bed net? | 1. Yes 2. No | | |  |
|  |  |  |  | 9.3 What do you do if your family member infected with malaria? | 1. visit HP  2. taking tradition medicine  3. dong no thing | | |  |
| **510** | | **First-aid emergency** | | 10.1 Have you ever had any accident? | 1. Yes  2. No | | |  |
|  |  |  |  | 10.2 If yes for the above q,did you do something on it the time of the accident to prevent further complication? | 1. Yes  2. No | | |  |
|  |  |  |  | 10.3 Have you gone to the nearest HP immediately? | 1. Yes  2. No | | |  |
| 1. **Family health** | | | | | | | | |
| **511** | | **Adolescent reproductive health** | | 11.1 is there suitable environment for adolescents and youthsreproductive health services when they want? | 1. Yes  2. No | |  | |
|  |  |  |  | 11.2 Have adolescents and youths used contraceptive methods freely when you want? | 1. Yes  2. No | |  | |
| **512** | | **Maternal and child health** | | 12.1 Have you visited health extension workers during pregnancy? | 1. Yes  2. No | |  | |
|  |  |  |  | 11.2 Have you visited health extension workers during labor | 1. Yes  2. No | |  | |
|  |  |  |  | 11.3 Have you visited health extension workers during post partum period? | 1. Yes  2. No | |  | |
| **513** | | **Nutrition** | | 13.1 have you fed your child breast milk exclusively before 6 month of age? | 1. Yes  2. No | |  | |
|  |  |  |  | 13.2 Have you visited health postin the last 12 months to get information about nutrition? | 1. Yes  2. No | |  | |
|  |  |  |  | 13.3 Have you provided supplementary food to your child after 6 month of age? | 1. Yes  2. No | |  | |
|  |  |  |  | 13.4 Have you feed yourself extra feeding during your pregnancy? | 1. Yes  2. No | |  | |
|  |  |  |  | 13.5 Have you feed yourself extra feeding during your post natal period? | 1. Yes  2. No | |  | |
| **514** | | **Immunization** | | 14.1 do you have under 5 children? | 1. Yes  2. No | |  | |
|  |  |  |  | 14.2 If yes for Q 14.1, are all fully immunized? | 1. Yes  2. No | |  | |
|  |  |  |  | 14.3 Have you received TT vaccine service in the past 12 months (for female) | 1. Yes  2. No | |  | |
| **515** | | **Family planning** | | 15.1 are there eligible persons who are using family planning service? | 1. Yes 2. No | |  | |
|  |  |  |  | 15.2 if yes for Q 15, are they using the service? |  | |  | |
|  |  |  |  | 15.3 if yes for Q 15.2, What type of contraceptive method have you ever used? | 1. Short term 2. Long term 3. Permanent method | |  | |
| 1. **Communication** | | | | | | | | |
| **516** | | Communication and health education | | **16.1** Have HEWs provided health education in the last 6 months? | 1. Yes 2. No | |  | |
|  |  |  |  | 16.2 Mention at least one thing that HEWs educated you | _____________ | |  | |

**መጠይቅ በአማርኛ**

**ክፍል 1፡ የማህበራዊ ና ኢኮኖሚያዊ ሁኔታን የሚዳስሱ መጠይቆች**

| ተ.ቁ | ጥያቄ | መልስ |
| --- | --- | --- |
| 101 | እድሜዎ ስንተት ነዉ? | ___________ዓመት |
| 102 | ፆታ | 1. ሴት 2. ወንድ |
| 103 | የጋብቻሁኔታዎ ምን ይመስላል? | 1. ያገባ/ች 2. ያላገባ/ች 3. ባልየሞተባት/በት 4. የፈታ/ች |
| 104 | የቤተሰብዎ ብዛት ስንት ነው? | __________________(በቁጥር) |
| 105 | ሐይማኖትዎምንድን ነው? | 1. ኦርቶዶክስ 2. ሙስሊም 3. ፕሮቴስታንት 4. ካቶሊክ 5. ሌላካለ ይጥቀሱ___________ |
| 106 | ብሔርዎ ምንድን ነው? | 1. አማራ 2. ኦሮሞ 3. ጉራጌ 4. ትግሬ 5. ሌላ ካለ ይጠቀሱ______ |
| 107 | የገቢ ምንጭ | 1. የእርሻ ስራ 2. ሌላ ______ |
| 108 | የትምህርት ደረጃዎ ሁኔታ? | 1. መደበኛ ት/ት ያልተማረ 2. የመጀመሪያ ደረጃ ት/ት ያጠናቀቀ 3. ሁለተኛ ደረጀ ት/ት ያጠናቀቀ 4. ኮሌጅ ወይም ከዛ በላይ ያጠናቀቀ |
| 110 | መኖሪያ ቦታ | 1. ገጠር 2. ከተማ |

ክፍል 2: ከጤና ኤክስቴንሽን ፓኬጅ አገልግሎት ጋር የተያያዙ መጠይቆች

| ተ.ቁ | ጥያቄ | መልስ |
| --- | --- | --- |
| 201 | ወደ ጤና ኬላ ለመሄድ የትራንሰፖርት አገልግሎት አለ? | 1. 1. አዎ2. የለም |
| 202 | ጤና ኬላ ሄደዉ ያዉቃሉ? | 1. አዎ 2. የለም |
| 203 | ለጥያቄ 202 መልስዎ አዎ ከሆነ ለምን አላማ? | 1. ለቅድመ ወሊድ ክትትል 2. ለቤተሰብ እቅድ አገልግሎት 3. ሌላ |
| 204 | ጤና ኬላዉ ከመኖሪያ ቤትዎ ምንያህል ይርቃል? | _____(በ ኪ.ሜ) |
| 205 | የጤና ኤክስቴንሽንባለሙያዋ/ዉ መስተንግዶ ምን ይመስላል? | 1.ትህትና የተላበሰ  2. ትህትና የጎደለዉ |
| 206 | የፈለጉትን አገልግሎት ከጤና ኬላዉ ሳያገኙ ተመልሰዉ ያዉቃሉ ? | 1. አዎ 2. የለም |
| 207 | ለጥያቄ 207 መልስዎ አዎ ከሆነ? ምክንያቱ ምንድነዉ | 1. አገልግሎት ስለሌለ  2. የጤና ኤክስቴንሽንባለሙያ በቦታዉ አለመገኘት |

**ክፍል 3: ከጤና ኤክስቴንሽን ፓኬጅ እዉቀት ና ሞዴል ጋር የተያያዙ መጠዪቆች**

| **ተ.ቁ** | **ጥያቄ** | **መልስ** |
| --- | --- | --- |
| 301 | ስለ ጤና ኤክስቴንሽን ፓኬጅ ሰምተው ያውቃሉ? | 1.አዎ  2.የለም |
| 302 | መፀዳጃቤት መጠቀም አንዱ የጤና ኤክሰቴንሽን ፓኬጅ አካል ነው፡፡ | 1.አዎ  2.የለም |
| 303 | ተገቢየሆነየደረቅእናፈሳሽቆሻሻአወጋገድ አንዱ የጤና ኤክሰቴንሽን ፓኬጅ አካል ነው፡፡ | 1.አዎ  2.የለም |
| 304 | የግልንጽኅናን መጠበቅአንዱ የጤና ኤክሰቴንሽን ፓኬጅ አካል ነው፡፡ | 1.አዎ  2.የለም |
| 305 | ንጹኅ የውሀአቅርቦትእናአጠቃቀምአንዱ የጤና ኤክሰቴንሽን ፓኬጅአካል ነው፡፡ | 1.አዎ  2.የለም |
| 306 | አግባብነትያለውቤትእናአካባቢ አያያዝ አንዱ የጤና ኤክሰቴንሽን ፓኬጅ አካል ነው፡፡ | 1.አዎ  2.የለም |
| 307 | የአይጦችናበቤትውስጥየሚኖሩጎጅነፍሳትቁጥጥርአንዱ የጤና ኤክሰቴንሽን ፓኬጅ አካል ነው፡፡ | 1.አዎ  2.የለም |
| 308 | የምግብንጽህናናአያያዝአንዱ የጤና ኤክሰቴንሽን ፓኬጅ አካል ነው፡፡ | 1.አዎ  2.የለም |
| 309 | ኤ.አይ.ቪ/ ኤ.ድስእናየሳነባነቀርሳአንዱ የጤና ኤክሰቴንሽን ፓኬጅ አካል ነው፡፡ | 1.አዎ  2.የለም |
| 310 | ወባንመከላከልእናመቆጣጠርአንዱ የጤና ኤክሰቴንሽን ፓኬጅአካል ነው፡፡ | 1.አዎ  2.የለም |
| 311 | ለድንገተኛአደጋዎችየመጀመሪያህክምናእርዳታአንዱ የጤና ኤክሰቴንሽን ፓኬጅአካል ነው፡፡ | 1.አዎ  2.የለም |
| 312 | የወጣቶችስነተዋለዶጤናአንዱ የጤና ኤክሰቴንሽን ፓኬጅአካል ነው፡፡ | 1.አዎ  2.የለም |
| 313 | የናቶችእናህጻናትጤናአንዱ የጤና ኤክሰቴንሽን ፓኬጅአካል ነው፡፡ | 1.አዎ  2.የለም |
| 314 | የናቶች ና ህጻናት ምግብ አንዱ የጤና ኤክሰቴንሽን ፓኬጅአካል ነው፡፡ | 1.አዎ  2.የለም |
| 315 | ክትባትአንዱ የጤና ኤክሰቴንሽን ፓኬጅአካል ነው፡፡ | 1.አዎ  2.የለም |
| 316 | የበተሰብእቅድአንዱ የጤና ኤክሰቴንሽን ፓኬጅአካል ነው፡፡ | 1.አዎ  2.የለም |
| 317 | የጤናትምህርትአንዱ የጤና ኤክሰቴንሽን ፓኬጅአካል ነው፡፡ | 1.አዎ  2.የለም |
| 318 | ከቤተሰብዎ መካከል በጤና ኤክስቴንሽን ፓኬጅ ሞዴል ስልጠና የተሳተፈ አለን? | 1.አዎ  2.የለም |
| 319 | በጤናኤክስቴንሽንፓኬጅሞዴልነትተመርቀዋል? | 1.አዎ  2.የለም |
| 320 | ለጥያቄ319 መልስዎአዎከሆነ, ምንያህልጊዜሆነዎት? | 1. 1–2 ዓመት  2. 3 ዓመት  3. ≥4 ዓመት |

**ክፍል 4: ከጤናኤክስቴንሽንፓኬጅአጠቃቀምጋርየተያያዙመጠዪቆች**

| **ሀ፡ንጽህናእናጽዳት** | | | | | | |
| --- | --- | --- | --- | --- | --- | --- |
| **ተ.ቁ** | | **ጥያቄ** | | | | **መልስ** |
| 401 | የቆሻሻአወጋገድ | | 1.1 መፀዳጃቤትአለዎት? | | 1. 1. አዎ 2. 2. የለም | |
|  |  |  | 1.2 የሚጠቀሙትመፀዳጃቤትምንአይነትነዉ? | | 1.የግል  2. የህዝብ | |
|  |  |  | 1.3መፀዳጃቤትዎንሁሌይጠቀሙበታል? | | 1. አዎ  2. የለም | |
|  |  |  | 1.4 ከመፀዳጃቤትዎጎንየእጅመታጠቢያአለ? | | 1. አዎ  2. የለም | |
| 402 | የግልንጽኅና | | 2.1 እጅዎትንምግብከማዘጋጀትዎ በፊት ይታጠባሉ? | | 1. አዎ  2. የለም | |
|  |  |  | 2.2 እጅዎትን ከምግብፊት ታጠባሉ? | | 1. አዎ  2. የለም | |
|  |  |  | 2.3 አብዛኛውንጊዜእጃችሁንለመታጠብምንትጠቀማላችሁ? | | 1. ሳሙና  2. አመድ  3. ምንምአንጠቀምም | |
| 403 | ተገቢየሆነየደረቅእናፈሳሽቆሻሻአወጋገድ | | 3.1 የቆሻሻመጣያችሁየትነው? | | 1. ግጣምየሌለውጉድጓድ 2. ግጣምያለውጉድጓድ 3. ማንኛውምቦታ   4. ማቃጠል  5.ለተፈጥሮማዳበሪያነትመጠቀም | |
|  |  |  | 3.2 ለፈሳሺቆሻሻማስወገጃየተዘጋጀቱቦአላችሁ? | | 1. አዎ  2. የለም | |
| 404 | የውሀአቅርቦትእናአጠቃቀም | | 4.1 የቤተሰባችሁየመጠጥውሃመገኛውየትነው? | | 1. ከቧንቧ  2. ከወንዝ  3. ሌላ------- | |
|  |  |  | 4.2 የምትጠቀሙበትንውሃአክማችሁ የምትጠቀሙት መንገድ አለ? | | 1. አዎ  2. የለም | |
|  |  | | 4.3 ለመጠጥየሚውለውንውሃለማስቀመጥየምትጠቀሙትእቃምንአይነትነው? | | 1.ቶፋ  2. ጀሪካ  3.ባልዲ  4.በርሚል | |
| 405 | አግባብነትያለውቤትእናአካባቢ | | 5.1 ኩሽናውከመኖሪቤታችሁየተለዬነውን? | | 1. አዎ  2. የለም | |
|  |  |  | 5.2 ኩሽናውውስጥየእቃመደርደሪያአላችሁ? | | 1. አዎ  2. የለም | |
|  |  | | 5.3 የመኖሪቤታችሁከቤትእንስሳትማደሪያየተለየነውን? | | 1. አዎ  2. የለም | |
| 406 | የአይጦችናበቤትውስጥየሚኖሩጎጅነፍሳትቁጥጥር | | 6.1 አይጦችንለመቆጣጠርየምትጠቀሙባቸውዘዴዎችምንድንናቸው? | | 1. የተፈጥሮጠላትንመጠቀም  2. የአይጥወጥመድመጠቀም  3. የአይጥመርዝመጠቀም | |
|  |  |  | 6.2 ቁንጫንናቅማልንስእንዴትትከላከላላችሁ? | | 1. ጸረነፍሳትመጠቀም  2. ልብስንመቀቀል  3.በእጅበመግደል | |
| 407 | የምግብንጽህናናአያያዝ | | 7.1 ምግብዎትንበጥንቃቄይሸፍናሉ? | | 1. አዎ  2. የለም | |
|  |  |  | 7.2 ምግቦችእንዳይበላሹየምታደርጉባቸውዘዴዎችአሉ? | | 1. አዎ  2. የለም | |
| **ለ፡ተላላፊበሽታዎችንመከላከልእናመቆጣጠር** | | | | | | |
| 408 | | ኤ.አይ.ቪ/ ኤ.ድስእናየሳነባነቀርሳ | | 8.ስለኤ.አይ.ቪ/ ኤ.ድስበጤናኤክስቴንሽንባለሙያተምራችሁታውቃላችሁ? | | 1. አዎ  2. የለም |
|  |  |  |  | 8.2 ስለኤ.አይ.ቪ/ ኤ.ድስምርመራየምክርአገልግሎትአግኝታችሁታውቃላችሁ? | | 1. አዎ  2. የለም |
|  |  |  |  | 8.3 የኤ.አይ.ቪ/ ኤ.ድስምርመራአድርጋችሁታውቃላችሁ? | | 1. አዎ  2. የለም |
|  |  |  |  | 8.4. ስለሳነባነቀርሳበጤናኤክስቴንሽንባለሙያተምራችሁታውቃላችሁ?? | | 1. አዎ  2. የለም |
|  |  |  |  | 8.5 ከሁለትሳምንትብላይየቆዬሳልቢያጋጥማችሁምንታደርጋላችሁ? | | 1. ጤናኬላእንሄዳለን  2. የባህልመድሃኒትእንወስዳለን  3. ምንምአናደርግም |
| 409 | | ወባንመከላከልእናመቆጣጠር | | 9.1 በአካባቢየችሁወባአለን? | | 1. አዎ  2. የለም |
|  |  |  |  | 9.2 መልስዎአዎከሆነለመከላከልአጎበር ትጠቀማላችሁ? | | 1. አዎ  2. የለም |
|  |  |  |  | 9.3 ከቤተሰብዎ መካከል በወባበሽታ የተጠቃቢኖርምንታደርጋላችሁ? | | 1. ጤናኬላእንሄዳለን  2. የባህልመድሃኒትእንወስዳለን  3. ምንምአናደርግም |
| 410 | | ለድንገተኛአደጋዎችየመጀመሪያህክምናእርዳታ | | 10.1 ድንገተኛአደጋደርሶብዎትያውቃል? | | 1. አዎ  2. የለም |
|  |  |  |  | 10.2 መልስዎአዎከሆነወደ ከፋ ድረጃ እንዳይደረስ ያደረጉት ነገር አለ? | | 1. አዎ  2. የለም |
|  |  |  |  | 10.3 ወዲያዉኑበቅርብወዳለጤናኬላሄደዉ ነበር? | | 1. አዎ  2. የለም |
| **ሐ፡የቤተሰብጤና** | | | | | | |
| 411 | | የወጣቶችስነተዋለዶጤና | | 11.1 ለወጣቶችስነተዋለዶጤና አገልግሎት የሚመች ቦታ አለ? | | 1. አዎ  2. የለም |
|  |  |  |  | 11.3 ወጣቶች የቤተሰብእቅድአገልግሎት በሚፍልጉ ጊዜ በነፃነት መጠቀም ይችላሉ? | | 1. አዎ  2. የለም |
| 412 | | የናቶችእናህጻናትጤና | | 12.1 በርግዝናዎወቅትጤናኤክስቴንሽንባለሙያአማክረዉነበር? | | 1. አዎ  2. የለም |
|  |  |  |  | 12.2 በዎልዱበትወቅትጤናኤክስቴንሽንባለሙያጋሄደዉነበር? | | 1. አዎ  2. የለም |
|  |  |  |  | 12.3 በድህረወሊድወቅትጤናኤክስቴንሽንባለሙያጋሄደዉነበር? | | 1. አዎ  2. የለም |
| 413 | | ስነምግብ | | 13.1 ልጅዎንካለምንምተጨማሪምግብለ 6 ወር  የእናትጡትወተትብቻአጥብተዋል? | | 1. አዎ  2. የለም |
|  |  |  |  | 13.2 ባለፉት 12 ዎራትዉሰጥከጤናኤክስቴንሽንባለሙያጋስለስነምግብአማክረዉያዉቃሉ? | | 1. አዎ  2. የለም |
|  |  |  |  | 13.3 ልጅዎንከ 6 ወርበላተጨማሪምግበአስጀምዋል? | | 1. አዎ  2. የለም |
|  |  |  |  | 13.4 በርግዘናዎዎቅትተጨማሪምግበይመገባሉ? | | 1. አዎ  2. የለም |
|  | |  | | 13.5 በድህረወሊድወቅትተጨማሪምግበይመገባሉ? | | 1. አዎ  2. የለም |
| 414 | | ክትባት | | 14.1 እድመያቸዉከ 5 አመትበታችህጻናትለዎት? | | 1. አዎ  2. የለም |
|  |  |  |  | 14.2 ለጥያቄቁጠር 14.1 አዎከሆነመልሰዎ፣ሁሉም መዉሰድ የሚጠበቅባቸዉን ሙሉክትባትዎስደዋል? | | 1. አዎ  2. የለም |
|  |  |  |  | 14.3 የመንጋጋቆልፍክትባትዎስደዉያዉቃሉ?(ለሴቶች ብቻ) | | 1. አዎ  2. የለም |
| 415 | | የበተሰብእቅድ | | 15.1 በቤትዎዉሰጥየቤተሰብእቅድአገልግሎትለመጠቀምየደረሰሰዉአለ? | | 1. አዎ   1. የለም |
|  |  |  |  | 15.2 ለጥያቄቁጠር 15.1 አዎከሆነመልሰዎ፣ያገልግሎቱተጠቃሚናቸዉ? | | 1. አዎ  2. የለም |
|  |  |  |  | 15.3 ለጥያቄቁጠር 15.2 አዎከሆነመልሰዎ፣የየትኛዉአይነትአገልግሎትተጠቃሚናቸዉ? | | 1.ለአጭርጊዜየሚያገለግሉትን  2. ለረጂምጊዜየሚያገለግሉትን  3.ብቋሚነትየሚያገለግሉትን |
| **ሐ፡መልእክት** | | | | | | |
| 416 | | መልዕክትናየጤናትምህርት | | 16.1 ባለፉት 6 ወራትዉሰጥጤናኤክስቴንሽንባለሙያዎችየጤናትምህርትሰጠዉያዉቃሉ? | | 1. አዎ  2. የለም |
|  |  |  |  | 16.2 ጤናኤክስቴንሽንባለሙያዎችከስተማሩትዉስጥቢያንስአንድነገርይጥቀሱ | | __________ |
