# Supplementary material for: Health extension service utilization and associated factors in East Gojjam zone, Northwest Ethiopia: A community-based cross-sectional study
Source: PLoS One. 2021 Aug 19;16(8):e0256418. doi: 10.1371/journal.pone.0256418 (PMC8376075; doi:10.1371/journal.pone.0256418)
Supplement: Attachment — Submitted filename: Comments Health Extension Plos1_AK 12feb202112 Feb 2021.pdf [file pone.0256418.s003.pdf]

12 Feb 2021
Reviewing in Plos One
Title: “Health extension service utilization and associated factors in east Gojjam zone, North West Ethiopia: community based cross-sectional study”

Reviewer: Abear Kumie, Addis Ababa University

Section	Comments
**Overall**	The study is highly appreciated in view of contributing to knowledge in Health extension programme
**Abstract**	The **Introduction section** has provided the background information. Much advised to include the rationale or brief of the statement of the problem<br><br>**Methods section**:<br>• Consider including the number of stages<br>• Pls define at least the outcome of interest<br>• Results: pls elaborate “knowledge” of what?
**Introduction**	The background of the study is very well described. However, the motive leading to this study is not seen. Pls include the statement of the problem that has drawn the authors to have this study.
**Methods**	**Study area and period**<br>Pls include a brief description of the organization of the Health Extension service in East Gojam
	**Study design**<br>Advised to elaborate the mixed design
	**Study participants**<br>How the inclusion criteria were handled in reference to residence of a participant? Pls indicate as new comers biases the results.
	**Sample size:**<br>Read as sample size determination<br>There must be additional exercises in terms of calculating the sample size for possible accommodation of the size for the 2nd objective addressing associated factors.
	**Sampling techniques**<br>Include the number of stages<br>“The individual of the selected household was interviewed”: which individual must be stated: any member? The head? Gender?
	**Independent variables: Good to categorize them by themes**<br>Socio-demog: ….<br>Service related: ….
	**Operational definitions**<br>Pls elaborate the “Satisfactory knowledge”: on what items??

	There must be additional definitions for the variables listed in Table 3: proper safe and excreta disposal, Proper and safe solid and liquid management, Water supply and hygiene, personal hygiene, Proper and safe home environment , food hygiene, Insects and rodents control, etc. This must be annexed as a supplementary file or as a part of the article
	**Data collection**<br>The section must be created with an appropriate description: the tools, who collected the data, the variables (understood seen)
	**Data management**<br>The section must be created with an appropriate description. The focus must be on the composite factor for utilization and knowledge
	**Data processing and analysis**<br>1. The two sections, data processing/ management, and data analysis must be addressed separately.<br>2. "Variables having a P-value of less than 0.25 in the bivariate logistic regression analysis were fitted into the multivariable logistic regression model":<br>• Pls get a reference for the p<0.25 ?<br>• Any reason to use this very relaxed p-value?<br>3. Pls include how managed variables in reference to collinearity?
**Results**	Read as Results
	Service-related characteristics needs to be described in terms of clarity with clear language. It must be aligned with the description of variables in the methods section:<br>Transport access? good approach from the HEWs? Participants needed?<br><br>**Service-related characteristics**<br>Less than half (37%) of the study participants have transport access to the health post. Two hundred one (90.5%) the participants go good approach from HEWs. Again around 208 (93.7%) participants got participants needed (Table 2).
	Edit: Among the respondents, only 311(38.6): include as 38.6%
	Must check ALL COR indicated in Table 4 for consistency<br>Wrongly indicated

Utilized		Crude OR[95%CI]
No	Yes	
26	4	1
307	50	1.059(.354, 3.162)
354	65	1.194(.403,3.533)

Given that reference:
OR = (307/50)/(26/4) = 0.94 (not 1.059)
Use 2 digits
Indicate (.354, 3.162) as (0.35,3.16) just as an example

I believe there was a problem in coding the categories of the variable “HEP utilization” which must be Yes=1; No=0.

Discussion

Good the discussion is accommodating the two sp objectives. Pls address the following comments

1. The govt of Ethiopia claims 100% of coverage or accessibility of the HEP within < 5 kms. Assuming the HEP workers are scheduled to visit every household, it is a paradox that the utilization is 14.8%. This is unfortunate. The way how this result is compared with the existing one is just a speculation. It requires to be supported by tangible evidences. Reasons could be many
    - Organization of the HEP?
    - The distance as seen service-related characteristics?
    - Inadequate planned visit by the HEP workers
    - The frequency of visiting a HH by HEP worker per year?
    - The distance between HEP providers and Community centers??
    - HEP workers are too much loaded, and therefore, not visiting HHs?
    - May be the community has not good preference on HEP services?
    - Quality of the service?
    - Many others.

I am suggesting to perform a qualitative search to justify the low level of utilization as 14.8%, which enriches the acceptability of the results. No matter doing is retrospectively.

	Urban dwellers were 3.549 times more likely to<br>utilize the health extension packages as: “more likely to utilize” is not really the reflection of cross-sectional designs. Pls improve the statement.
Conclusion	The conclusion is a heavy blow to the HEP. The HEP provider cannot appreciate this result unless good reasons are indicated. The factors indicated in the results section (residence, HH visiting HP, Training) are not adequate to indicate feasible interventions.<br><br>“Strict home to home visit especially in the rural area may increase the health extension package utilization”: I guess this was not supported by the results. The results simply showed respondents visiting the Health Posts and not vice versa.
**Limitations**	There must be a description on this section<br><br>• What is the reason to find in a result section 96.1% graduated Model given the sampling criteria with HHs graduated of HEP?<br>• Any other limitation?
**Reviewer Decision**	Major revision is needed in terms of addressing the comments that are indicated in the methods section, results and discussion section.
