# Supplementary material for: Health extension service utilization and associated factors in East Gojjam zone, Northwest Ethiopia: A community-based cross-sectional study
Source: PLoS One. 2021 Aug 19;16(8):e0256418. doi: 10.1371/journal.pone.0256418 (PMC8376075; doi:10.1371/journal.pone.0256418)
Supplement: Attachment — Submitted filename: Response to Reviewers.docx [file pone.0256418.s005.docx]

1. The gap was described as "38% of children under the age of five years suffered from stunting due to malnutrition; the Amhara region accounts for the highest proportion (46%) and the prevalence of water-borne and water-washed diseases was high. Under 5 mortality 30%, maternal mortality 412 per100, 000 live births, and unmet need for family planning are 22%. These figures showed the gaps and more gap description is not needed.
2. The Ethiopian government recommended that HEP coverage be 100%. So based on that recommendation, the finding from our study is low utilization.
3. I included participants in age >=18 years because they can give full information about the needed data and can give their consent.
4. Calculating the sample size based on the second objective is not mandatory since the sample size calculated from prevalence is large enough.
5. The number of kebeles and woredas is determined by 20%(there is a rule to take 20% and above ) of the total kebeles and woredas.
6. I used 0.25 as a variable screening to increase the chance of the variables (the number of variables) in the model. As a rule, our collage uses this cut point.
7. Multicolleanity among explanatory variables was checked.
8. The assumptions of the binary logistic regression model were checked.
9. The backward selection method was used.
10. Sometimes the response rate could be 100%. In this study, the participants were volunteers and all respondents gave their responses.
11. Since the data can be presented differently, we can display it in text form. Only presenting on the table is not an obligation.
12. Other variables effect controlled in the regression model.
13. When we justify the possible reason in the discussion part, we can justify it in different ways and citation may or may not be mandatory.
14. Since the study is a cross-sectional study design the limitation is known and taking about strength and limitation in a cross-sectional study has no value.
15. Since the outcome of interest is defined in the introduction part of the abstract, there is no need to define the method of the abstract.
16. Since the overall HEP(outcome variable) is defined and each HEP is not a factor/ outcome variable, there is no need to define each.
