# Supplementary material for: Health extension service utilization and associated factors in East Gojjam zone, Northwest Ethiopia: A community-based cross-sectional study
Source: PLoS One. 2021 Aug 19;16(8):e0256418. doi: 10.1371/journal.pone.0256418 (PMC8376075; doi:10.1371/journal.pone.0256418)
Supplement: Attachment — Submitted filename: Response to Reviewers.docx [file pone.0256418.s006.docx]

1. Dear reviewers, I would like to forward my gratitude to you for your constructive comments. I have learnt many things from your comments.
2. Dear 4^th^ reviewer, I have accepted your comments and some correction are done based on your comments.
