# Supplementary material for: Health extension service utilization and associated factors in East Gojjam zone, Northwest Ethiopia: A community-based cross-sectional study
Source: PLoS One. 2021 Aug 19;16(8):e0256418. doi: 10.1371/journal.pone.0256418 (PMC8376075; doi:10.1371/journal.pone.0256418)
Supplement: Attachment — Submitted filename: Response to Reviewers.docx [file pone.0256418.s007.docx]

Dear reviewers

**Thanks your guidance and comments**

- We have addressed all comments.
