# Supplementary material for: Health extension service utilization and associated factors in East Gojjam zone, Northwest Ethiopia: A community-based cross-sectional study
Source: PLoS One. 2021 Aug 19;16(8):e0256418. doi: 10.1371/journal.pone.0256418 (PMC8376075; doi:10.1371/journal.pone.0256418)
Supplement: Attachment — Submitted filename: Reviewer comment to author.docx [file pone.0256418.s004.docx]

**Reviewer comment to author’s**

Title: - Health extension service utilization and associated factors in east Gojjam zone, North

West Ethiopia: community based cross-sectional study.

Thank you very much editor for give me a chance to review this interesting manuscript. I thank you for authors for your effort and interesting work. I think this work adds value for the scientific community and the community in which the study done after the following issues addressed.

**General comment**

Before submitted any manuscript to journal you should give line number and follow the guideline of the journal. This manuscript does not have line number and it is difficult to give line-by-line comments. Anyways my comment given each session of the manuscript find it and address it.

**Abstract**

**Introduction**

The introduction section of the abstract does not show the gap. What is the reason to study this research? It needs rewriting explain the gap and reason to conduct this study needed. Before you talk about the significance of the study, you should tell the gap.

**Method**

The method session needs some modification. The model fitness and include the bivariable analysis variable selection p-value

**Result**

The abbreviation HEW did not explained before but written their it not write. The abstract session should be free and abbreviation

Conclusion: - In the first sentence, you said, “The magnitude of health extension service utilization was low.” What is your cutting point to say high/low? Such types of conclusion is blind and not informative.

**Body of the manuscript**

**Introduction**

In your introduction session, I did not see any factors associated with health extension service utilization what previous scholars explore. It needs one paragraph the reviews the associated factors of health extension service utilization in Ethiopia. Include it in your next revision part.

**Method**

In the study design session said” Community based mixed cross-sectional study design” what does it mean? I did not see any qualitative part. I need a justification such types of study design.

What your justification in your study participants session you included participants in age >=18 years?.

In your sample size calculation formula you only calculated for prevalence of health extension service utilization. Why not calculate for factors. You are expected to calculate for both the magnitude and the factor then you will take sample size which large. Why that not done?

In your sampling technique from 5/21 woredas why only 5? Justify it. Again the seleceted kebeles from total woredas ? how determine the number of kebele for each woreda? Justfy it.

In your data processing and analysis you used 0.25 as a variable screening but there is no any justification. Justify why that?

The model fitness issue is not presented. Why? Justify it. Include in the revision part

Did you check multicolleanity among explanatory variables? if not why justify it.

Did you check assumptions of binary logistic regression model?

What variable selection method you used? Forward/backward/stepwise?

**Result**

In the result section it said that “giving a response rate of 100%.” How it could be? Unbelievable result. justify it.

In the “Knowledge and modeling related characters” has no table citation? Why?

In the model interpretation session you said that “After controlling the effect of other variables

with binary logistic regression, educational status, residence, knowledge, health postvisit,

home visited by HEW, and model family training continued to be significantly associated with utilization of HEPs (P-values<0.25).” how other variables effect controlled in the bivariable analysis part? Justify it.

In the final model result interpretation section “After controlling the effect of other variables with multivariable logistic regressionanalysis, residence [AOR: 3.549(95% CI: 1.99, 6.328)], knowledge [AOR: 1.844(95%CI: 1.216, 2.796)] health post-visit [AOR: 1.625(95% CI: 1.054, 2.504)], home visited by HEW [AOR: 1.676(95% CI: 1.025,2.740)] and Graduated from model family training [AOR: 2.104(95% CI: 1.377,3.215)] were significant factors for health extension

package utilization (Table 4 ).”

Such type of writing should be in the abstract session, you are expected interpret the result explicitly. Correct it during the revision.

**Discussion**

I found your result and discussion section mixed. You should separate them. If the result is correctly, interpret in the result session no need to repeat it in the discussion session.

In the discussion section your speculation needs citation example “The other possible reason may be a limitation in basic infrastructures in rural communities relative to the Gulelle sub-city administration community.” Need to be supported by evidence…..works for all discussion session.

I did not see any strength and limitation of the study. Why ? justify it?

**Conclusion**

The conclusion and recommendation should be based on your findings.

**Declaration session**

No Abbreviations and Acronyms

Totally you did not follow the journal guideline please follow it and correct it accordingly**.**
